# Infant Lung Function in Cystic Fibrosis: A Real‐World Study

**DOI:** 10.1002/ppul.71117

**Published:** 2025-05-06

**Authors:** Michele Arigliani, Sidrah Chaudhry, Rossa Brugha, Ranjan Suri, Paul Aurora

**Affiliations:** ^1^ Paediatric Respiratory Unit Great Ormond Street Hospital for Children NHS Foundation Trust London UK; ^2^ Infection, Immunity and Inflammation Research and Teaching Department UCL Great Ormond Street Institute of Child Health (UCL GOS ICH) London UK

**Keywords:** cystic fibrosis, multiple breath washout, pediatrics, real‐world evidence, respiratory function tests

## Abstract

**Background:**

Previous research showed that lung function abnormalities are common in infants with cystic fibrosis (IwCF) but real‐world data are missing.

**Methods:**

This single‐center retrospective study analyzed infant lung function results from IwCF born in 2012–2018. The tests were conducted at Great Ormond Street Hospital, London, as part of routine care at 3 months, 1 year, and 2 years of age. Z‐scores for SF_6_ Lung Clearance Index (zLCI), plethysmographic FRC (zFRC_pleth_) and FEV_0.5_ were derived. Microbiology and antibiotics prescription from 3 months before lung function assessments, up to the closest medical review following the lung function encounter, were analyzed, along with changes in management advised by the physician.

**Results:**

A total of 126 lung function encounters (*n* = 43 at 3 months, 46 at 1 year, 37 at 2 years) from 60 IwCF were included. LCI was abnormal (zLCI > 1.96) in 31% (12/39) of 3‐month‐olds (mean± zLCI 1.21 ± 1.08), 28% (12/43) of 1‐year‐olds and 19% (7/36) of 2‐year‐olds (mean± zLCI 1.13 ± 1.10). Among 74 cases with recent positive microbiology or abnormal chest findings at medical review, 100% (31/31) of those with abnormal lung function and 86% (37/43) of those with normal lung function (*p* = 0.04) had a recent antibiotic prescription or a change in clinical management. Conversely, in encounters with abnormal lung function but normal clinical findings, management changes occurred in only 12% (2/16) of cases.

**Conclusion:**

In this real‐word cohort of IwCF, clinical management was mainly influenced by clinical findings and only marginally by abnormal lung function (elevated FRC or LCI).

## 1 Introduction

Chronic lung disease in cystic fibrosis (CF) starts very early in life [1] and previous studies using infant lung function (LF) showed that abnormalities can be present in a minority of patients already by 3 months of age [2, 3]. The Lung Clearance Index (LCI), a marker of early CF lung disease [4], is abnormal in 15%–20% of infants with this condition [5, 6] and, if elevated at the age of 2 years, tends to remain abnormal during the pre‐school years [7]. However, abnormal LF results, including LCI values, are mostly mild and transient in infants with CF [5]. This information has been obtained from controlled studies, and the utility of regular infant LF testing in a real‐world clinical setting is uncertain.

At Great Ormond Street Hospital (GOSH), London, UK, LF testing was performed routinely in infants with CF (IwCF) between 2010 and 2020. The objective of this retrospective analysis was to (a) evaluate success rates and results of clinically ordered infant LF tests; and (b) to relate LF results and clinical information of those infants to management decisions that occurred around the time of infant LF.

We hypothesized that children with abnormal infant LF tests would have had more frequent changes in management at around the time of the infant LF datapoint.

## 2 Methods

This was a single‐center retrospective cohort analysis and received approval as a Service Evaluation by the GOSH Clinical Audit department (approved in May 2022). Given the design of retrospective audit analysis and the use of anonymized data in the final analysis, informed consent was not required. All IwCF under follow‐up at GOSH, London, born between 2012 and 2018, were eligible. The clinical management of IwCF at our center generally follows internal pediatric CF guidelines that are based on national and international standards [8, 9] and are updated approximately every 5 years. Additionally, until 2020 IwCF routinely underwent LF assessments at approximately, 3 months, 1 year and 2 years of age when clinically stable. The majority of children also underwent routine surveillance bronchoscopy and bronchoalveolar lavage (BAL) at 1 year of age. Each test session included: (a) Sulfur hexafluoride (SF_6_) multiple breath washout (MBW) for LCI; (b) body plethysmography for functional residual capacity (FRCpleth); (c) the raised volume rapid thoracic compression (RVRTC) technique for Forced Expiratory Volume at 0.5 s ‐FEV 0._5_‐) (in this order). The tests were perfomed by senior pediatric physiologists at the GOSH LF laboratory, using methodology and equipment as previously reported in the London Cystic Fibrosis Collaboration (LCFC) infant cohorts [5, 10]. Before each test session, oral sedation (chloral hydrate 60–100 mg·kg^−1^; maximum 1000 mg) was administered. Lung function results were converted to z‐scores using published reference equations derived from healthy infants and obtained with the same equipment and protocols [11, 12, 13]. Limits of normal were < −1.96 z‐scores for FEV_0.5_ and > 1.96 z‐scores for LCI and FRC_pleth_, based on the reference population.

During this period there was no standard operating procedure on how clinicians should act upon abnormal LF results in infants or in older children. Results were interpreted in the context of the overall clinical status on a case‐by‐case basis by a senior clinician after discussion with the multidisciplinary team.

Patients who did not have a LF result available from the first 2 years of life were excluded from the analysis.

Two investigators (MA and SC) reviewed the clinical documentation concerning the period around between each LF data point, as well as medical correspondence from the outpatient clinic closest in time to each LF encounter. This identified: (a) positive lower airway bacterial culture (cough swab, sputum or bronchoalveolar lavage ‐BAL‐, except for Enterobacteriaceae and commensals) that required an antibiotic course from 3 months before the LF data point up to the closest clinical encounter following each LF data point; (b) the presence of abnormal chest signs or symptoms or a history of cough for most days in the last 3 week, at the time of the closest medical review following a LF data point; (c) any documented change in clinical management, such as additional investigations ordered or adjustments in medication, advised at the closest medical review following a LF data point.

Information on airway microbiology results from the time of the previous infant LF datapoint (or before 3 months of life) was also collected. Clinical data were extracted from the documentation uploaded on the hospital's electronic health record (EPIC Systems, Verona, United States) for the period 2012–2018 and directly from the hospital's software starting from 2018, when the software use was introduced in our hospital.

Group comparisons were performed using Fisher exact test as appropriate. Analysis was performed using Prism version 10.2.3 (GraphPad Software).

## 3 Results

The GOSH cohort of IwCF born in 2012–2018 comprised 103 subjects. Of these, 59% (60 of 103, 30 males and 30 females) underwent at least one LF assessment by the age of approximately 2 years, for a total of 126 LF encounters. The final study cohort included three subjects born preterm (one at 27 weeks of gestation and two at 35 weeks), 48 subjects (80%) homozygous or heterozygous for p.Phe508del, 10 subjects (17%) with a history of meconium ileus and 5 subjects (8%) who were pancreatic sufficient. Clinical details of the CF infants in relation to test occasions at 1 year and 2 years of age are described in Table 1.

**TABLE 1:** Clinical characteristics of Infants with cystic fibrosis at the lung function test encounter at 1 year and 2 years of age.

	1‐year‐old (n. 46)^a^	2‐year‐old (n. 37)
Additional treatment in the preceding year		
rhDNase^b^	3 (6%)	5 (13%)
IV antibiotics^c^	20 (43%)	12 (32%)
≥ 3 courses of oral antibiotics	23 (50%)	21 (57%)
Gastroesophageal reflux disease treatment^d^	23 (50%)	19 (51%)
Most relevant lower airway microbiology in the year before lung function encounter^e^		
*Pseudomonas aeruginosa*	10 (22%)^f^	8 (22%)^f^
*Staphylococcus aureus*	11 (24%)^g^	5 (13%)^g^
*Haemophilus influenzae*	17 (37%)	14 (38%)

a 29/46 subjects also had surveillance bronchoalveolar lavage at 1 year of age.

b recombinant human DNase started as long‐term therapy in the 12 months before the lung function encounter.

^c^ At least one emergency or planned admission for intravenous antibiotics in the 12 months before the lung function encounter.

^d^ Proton pump inhibitors.

^e^ Number (% of tot) of patients who had at least one positive bacterial culture from bronchoalveolar lavage or surveillance sputum/cough swabs samples.

^f^ 4/18 children who had *Pseudomonas aeruginosa* at 1‐year‐old or 2‐year‐old lung function encounter also had *Haemophilus influenzae* and 1/18 also had *Staphylococcus aureus.*

^g^ 4/16 children who had *Staphylococcus* aureus at 1‐year‐old or 2‐year‐old lung function encounter also had *Haemophilus influenzae.*

There were 126 LF encounters from 60 IwCF. The failure rate was lowest for LCI at 4% (5/126 attempts), intermediate for FRC_pleth_ at 21% (25/118 attempts), and highest for FEV_0.5_ at 35% (38/108 attempts).

Cross‐sectional LF results of the CF cohort at ∼3 months of age (n. 43), 1 year (n. 46) and 2 years (n. 37) are presented in Table 2.

**TABLE 2:** Cross‐sectional data of anthropometry and lung function at ∼3 months, 1 year, and 2 years of age in the GOSH cystic fibrosis cohort born 2012–2018.

	3 months	1 year‐old	2 years‐old
	n. 43	n. 46	n. 37
Age at test (months), median (IQR)	3.3 (3.0–4.1)	11.7 (11.1–12.8)	23.1 (22.4–23.9)
zHeight [14]	−0.17 ± 1.01	0.27 ± 1.20	0.04 ± 1.19
zBMI [14]	−0.85 ± 0.97	−0.06 ± 1.06	0.38 ± 1.10
LCI n.	39	43	36
LCI absolute value	8.05 ± 0.88	8.05 ± 1.03	7.58 ± 0.78
zLCI [11]	1.21 ± 1.08	1.52 ± 1.43	1.13 ± 1.10
FRC_pleth_ n.	34	43	23
zFRC_pleth_ [13]	0.77 ± 1.00	−0.33 ± 1.08	1.23 ± 1.57
RVRTC n.	26	22	7
zFEV_0.5_ [12]	−0.08 ± 1.40	−0.02 ± 1.07	0.24 ± 0.38

*Note:* Data are presented as mean ± SD, unless otherwise stated.

Abbreviations: BMI, body mass index; FEV_0.5_, forced expiratory volume in 0.5 s; FRCpleth, plethysmographic functional residual capacity; IQR, Interquartile range; LCI, lung clearance index; RVRTC, raised volume rapid thoracic compression; Z, z‐score;

LCI was most frequently abnormal, with values > 1.96 z‐scores seen in 31% (12/39) of the cohort at 3 months of age and in 19% (7/36) at 2 years of age (Table 3). Noticeably, none of the subjects who had LCI recorded at all three timepoints (n. 18), showed consistently increased ventilation inhomogeneity over the time between 3 months and 2 years of age (Table 3). Gas trapping (FRC_pleth_) had a progressively increasing frequency from 12% (4/12) at 3 months, to 17% (8/17) at 1 year, to 37% (9/24)by 2 years of age. FEV_0.5_ was abnormal only in 4% (2/55) of cases (Table 3).

**TABLE 3:** Number of tests obtained and proportion of abnormal results in infants with cystic fibrosis at each test occasion between 3 months and 2 years of age.

	3 months n. 43	1 year n. 46	2 years n. 37	3 m and 1 yr n. 33	3 m and 2 yr n. 27	1 yr and 2 yr n. 31	3 m, 1 yr, 2 yr n. 31
Total LCI	39	43	36	27	18	19	18
LCI > 1.96 z‐scores [11] n. (%)	12 (31%)	12 (28%)	7 (19%)	4 (15%)	1 (4%)	2 (10%)	0
Total FRC_pleth_	34	43	24	25	17	18	13
FRC_pleth_ > 1.96 z‐scores [13] n. (%)	4 (12%)	8 (17%)	9 (37%)	2 (8%)	2 (7%)	2 (18%)	1 (8%)
Total FEV_0.5_	26	22	7	11	7	9	1 (10%)
FEV_0.5_ < −1.96 z‐scores [12] n. (%)	2 (8%)	0	0	0	0	0	0
All lung function tests obtained	20 (51%)	19 (41%)	5 (35%)	7 (21%)	7 (26%)	5 (16%)	1 (3%)

Abbreviations: FEV0.5, forced expiratory volume in 0.5 s; FRC_pleth_, plethysmographic functional residual capacity; LCI, lung clearance index.

**FIGURE 1:**
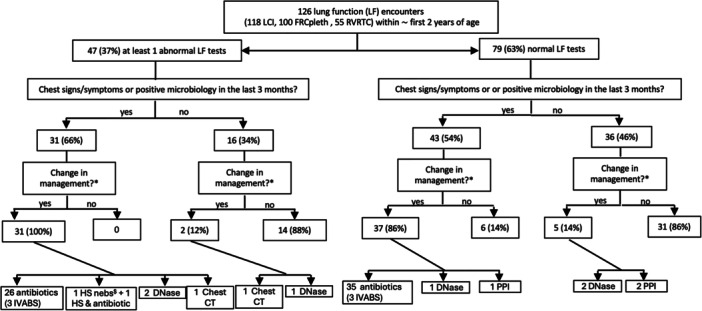
Changes in clinical management around the time of infant lung function tests results in infants with cystic fibrosis. *Antibiotics prescriptions occurred between 3 months before lung function and the medical review in clinic closest to the lung function datapoint. ^$^One subject was started on DNAse due to symptoms report and the other due to recent bronchoscopy findings. ^£^Started on PPI due to gastroesophageal symptoms report. Legend: CT, Computed tomography; FRC_pleth_, plethysmographic functional residual capacity; HS nebs, Hypertonic saline nebulization; IVABS, intravenous antibiotics; LCI, lung clearance index; PPI: proton pump inhibitors; RVRTC, raised volume rapid thoracic compression;

Details regarding changes in clinical management around the time of LF results (e.g., additional investigations ordered or new medication started) are provided in Figure 1.

In summary, 47 out of 126 encounters had at least one abnormal LF test (37%). This subgroup of IwCF tended to have more frequent abnormal chest findings at medical review or recent positive airway bacterial microbiology compared to those with normal LF (31/47, 66% vs. 43/79, 54%, P = N.S.) (Figure 1). A recent course of antibiotic or a change in clinical management following medical review occurred in 100% of 31 IwCF with abnormal LF results and abnormal chest findings at review or recent positive airway bacterial microbiology. Specifically, 26 IwCF had antibiotics (including 3 with intravenous antibiotics), while, following medical review, one was started on regular hypertonic saline (HS) nebulisation, one had HS and an antibiotic prescribed, one had a chest CT ordered and two were started on rhDNase. In contrast, a recent course of antibiotic or a change in management following medical review occurred in 86% (37/43) of IwCF with normal LF results but abnormal clinical findings (*p* = 0.04). Among these, 35 had antibiotics, whereas following medical review one was started on rhDNase and one was started on a proton pump inhibitor (PPI) for gastroesophageal reflux (GER) symptoms (Figure 1).

Only two out of 16 IwCF (12%) with abnormal LF but normal clinical findings had a change in clinical management; one underwent a chest CT and the other was started on rhDNase (Figure 1).

Among potential risk factors for abnormal LF, a higher frequency of GER treatment at 3 months of age was observed in IwCF with abnormal LCI (7 out of 12, 58%), compared to those with normal LCI (3 out of 11, 27%) (*p* = 0.003). No other statistically significant differences were observed in the variables listed in Table 1 across each LF data point between IwCF with high (> 1.96 z‐scores) versus normal LCI (Table 4).

**TABLE 4:** Clinical characteristics of infants with cystic fibrosis with abnormal versus normal Lung Clearance Index at each data point.

	3‐month‐old		1‐year‐old		2‐year‐old	
	High LCI n. 12	Normal LCI n. 27	High LCI n. 12	Normal LCI n. 31	High LCI n. 7	Normal LCI n. 29
Additional treatment in the last year/months						
rhDNase	—		2 (16%)	0	1 (14%)	4 (14%)
IV antibiotics	1 (8%)	6 (22%)	6 (50%)	10 (32%)	3 (42%)	9 (31%)
≥ 3 courses of oral antibiotics between 1 and 2 yrs or ≥ 1 antibiotic by 3 m.o.	5 (41%)	14 (51%)	6 (50%)	12 (39%)	3 (42%)	19 (65%)
Gastroesophageal reflux disease treatment	7 (58%)*	3 (11%)	6 (50%)	13 (42%)	3 (42%)	16 (55%)
Lower airway microbiology in the last year						
*Pseudomonas aeruginosa*	0	1	3 (25%)	7 (23%)	1 (15%)	9 (31%)
*Staphylococcus aureus*	2 (17%)	5 (18%)	3 (25%)	8 (25%)	1 (15%)	4 (14%)
*Haemophilus influenzae*	0	2 (7%)	2 (16%)	15 (48%)	3 (43%)	11 (37%)

Abbreviations: IV, intravenous; LCI, Lung Clearance Index; rhDNase, recombinant human DNase.

* *p* = < 0.05.

## 4 Discussion

In this real‐world evidence (RWE) analysis of longitudinal lung function changes in IwCF, we found that abnormal LF was predominantly mild and transient between the ages of 3 months and 2 years. Changes in clinical management around the time of the LF encounters (but not necessarily after these) were more common in cases where abnormal LF results was accompanied by abnormal clinical findings (e.g., abnormal chest signs/symptoms at review or recent positive lower airway microbiology), suggesting that, in many instances, infant LF assisted the decision making of the clinicians. In contrast, an abnormal LF without corresponding clinical concerns rarely led to any change in management. This finding likely reflects clinicians’ uncertainty regarding whether minor abnormalities in infant LF are definitely markers of early CF lung disease or whether they can sometimes resolve without intervention. In fact, despite LCI has been successfully used as an endpoint in a trial assessing the effectiveness of preventive HS nebulization in IwCF [15], longitudinal studies have yet to establish a clear association between abnormal infant LF results and structural lung disease, as assessed by chest CT [6, 16], or poorer long‐term clinical outcomes.

In our study, approximately 30% of IwCF had an abnormal LCI at 3 months and 1 year of age (Table 3). This frequency is higher than reported in earlier studies utilizing SF_6_ MBW, which found rates between 15% and 22% in research cohorts from the UK and Australia [5, 17], but lower than the 44% frequency observed in a recent CF infant cohort from northern Europe [18]. Consistent with previous data from LCFC, abnormal LF results in IwCF were predominantly transient throughout infancy, with a tendency towards reduced ventilation inhomogeneity and increased gas trapping by 2 years of age [5]. It is noteworthy, however, that in this age group, an elevated FRC_pleth_ did not correlate with gas trapping on chest CT [16]. A previous cohort study of over 100 IwCF from Australia found that infants with positive BAL bacterial cultures at 3 months, 1 year or 2 year of age [19], had a worsening LCI during the first 2 years of life. In contrast, our study did not find an association between positive airway microbiology results and a higher frequency of abnormal LCI (Table 4). However, the small sample size in our cohort and the absence of serial BAL cultures throughout infancy strongly limits the generalizability of this finding. More recently, *Sandvik* et al observed a worsening trajectory of ventilation inhomogeneity from early infancy to preschool age in a nationwide Danish cohort study of 59 people with CF (PwCF) [20]. Lung function deterioration was more pronounced in those who had at least one positive lower airway culture for *Pseudomonas Aeruginosa*, but was partially alleviated by starting lumacaftor/ivacaftor at 2 years of age [20]. In contrast, we did not identify a longitudinal trend in lung function in the IwCF of our cohort, likely due to the limited observation period, which ended at 24 months of life.

Regarding the influence of LF results on clinical decision making in PwCF, no comparative study have been conducted in IwCF. Limited evidence is available for preschool and school‐age PwCF, where LCI is a well‐established marker of early CF lung disease [21, 22], anticipating future lung function abnormalities [23, 24, 25] and structural lung damage [26]. A pilot randomized controlled trial investigated whether performing a BAL based on an LCI increase of at least 1 unit in 3‐monthly MBWs over a 2 years could improve clinical outcomes or increase pathogens detection in school‐age PwCF [27]. The study found no significant benefit in the intervention group. However, due to the limited sample size, the findings may not be generalizable. Additionally, a recent clinical vignette survey of 62 CF physicians evaluated the impact of LCI results on clinical decision‐making. In this study, physicians were presented with hypothetical clinical scenarios of children with CF [28]. When LCI results were communicated after a clinical management decision had already been made, decisions changed in 18% of cases, were supported by LCI in 57%, and remained unchanged in 24% [28]. Although our analysis is not directly comparable due to differences in methodology and patients age groups, our findings align with this trend. Specifically, we observed more frequent changes in management around the time of the LF encounter in IwCF with abnormal versus normal LF results (66% vs. 54%, P = NS). This is consistent with the findings of *Perrem* et al, suggesting that in a real‐world setting, LF results play a role in supporting the decision making for most CF patients.

Regarding the impact on clinical decision making of spirometry results, pre‐CFTR‐modulators real‐world data showed that around 25% of people with CF (PwCF) failed to recover baseline spirometry within 6 months from a PEx [29]. Despite this, up to 30% of PwCF with an acute FEV_1_ drop ≥ 10 percentage predicted (pp) may not receive antibiotic treatment [30]. The adoption of a PEx signal based on FEV_1_ pp acute decline, combined with a management algorithm that considers the extent of FEV_1_ pp decline and the presence of symptoms, markedly increased the proportion of PwCF receiving interventions due to abnormal LF results [31]. Whether these changes in clinical management based on LF results translate to improved clinical outcomes, however, remains unclear.

The failure rate of FEV_0.5_ using RVRTC in this real‐world cohort (35%) was much higher than in previous research cohorts tested in the same laboratory, where FEV_0.5_ was obtained in the great majority of IwCF [5, 17]. Given that the seniority level of the physiologists conducting the tests, the light sedation used and the order of the LF tested performed were similar between the research and real‐world cohorts, the primary reason for this marked difference is likely the tighter time constraints of clinically ordered tests, which limited the number of attempts, especially if the infant woke up during the session.

Interestingly, the great majority IwCF who achieved an acceptable FEV_0.5_ in our real‐world cohort did not exhibit airflow limitation. Their mean FEV_0.5_ z‐score was higher by 0.5–1 z‐scores at each data point compared to those in the most recent LCFC infant cohort [5], which included IwCF born 2–9 years before those in the current cohort. We hypothesize that improvements in the quality of care for IwCF at our center during this period, particularly, the optimization of the clinical pathway for infants with positive neonatal screening (e.g., reducing the time to diagnosis confirmation and first visit at the CF center) may have contributed to these results.

We could not identify specific risk factors associated with abnormal infant LF in our CF cohort. There was a high frequency of GER treatment in those with abnormal LCI at 3 months of age. These children had been started on PPI empirically, only based on symptoms report and according to the clinician judgment,. People with CF (PwCF) are at higher risk of GER [32] and, although GER is very common in healthy infants and typically does not require treatment [33], symptomatic IwCF are often started empirically on PPI due to concerns about the risk of aspiration [34]. In the absence of objective GER assessments in this cohort, one could speculate that a higher LCI in IwCF receiving PPI treatment may reflect some degree of comorbid aspiration lung disease, contributing to increased ventilation inhomogeneity. However, differences in LCI between IwCF on PPI and the rest of the cohort was no longer apparent at the 1‐year and 2‐year time points, suggesting that the association between GER treatment and increased LCI at 3 months of age may have been coincidental.

The main limitation of the study is its retrospective nature, which may have introduced potential biases impacting the accuracy of the data, particularly regarding the determinants of changes in clinical management (e.g., determinants that were not documented would have been missed). We were unable to establish the exact timing of antibiotic prescriptions around each LF data point in children with positive airway microbiology in the previous 3 months, limiting our ability to estimate the impact of the infant LF test results on clinical management. Additionally, the study was not powered to detect differences in clinical outcomes between patients with normal and abnormal LF.

The main strength is that this is among the first reports presenting longitudinal lung function real‐world data in IwCF.

In conclusion, this study showed that in a real‐world cohort of infants with CF, abnormal infant LF results (elevated FRC or LCI) were mostly mild and transient until 2 years of age. Clinical management was mainly influenced by clinical findings and to a lesser extent by abnormal lung function.

Future studies should elucidate the prognostic implications, if any, of abnormal infant LF results in CF patients. This clarification will be pivotal in determining whether routine performance of these assessments on clinical grounds is justified.

## Author Contributions

**Michele Arigliani:** conceptualization, investigation, writing – original draft, methodology, formal analysis, data curation. **Sidrah Chaudhry:** conceptualization, methodology, investigation, writing – review and editing, supervision. **Rossa Brugha:** conceptualization, writing – review and editing; supervision. **Ranjan Suri:** conceptualization, writing – review and editing, supervision. **Paul Aurora:** conceptualization, writing – review and editing, supervision.

## Disclosure

The authors have nothing to report.

## Conflicts of Interest

The authors declare no conflicts of interest.

## Data Availability

The data that support the findings of this study are available from the corresponding author upon reasonable request.
